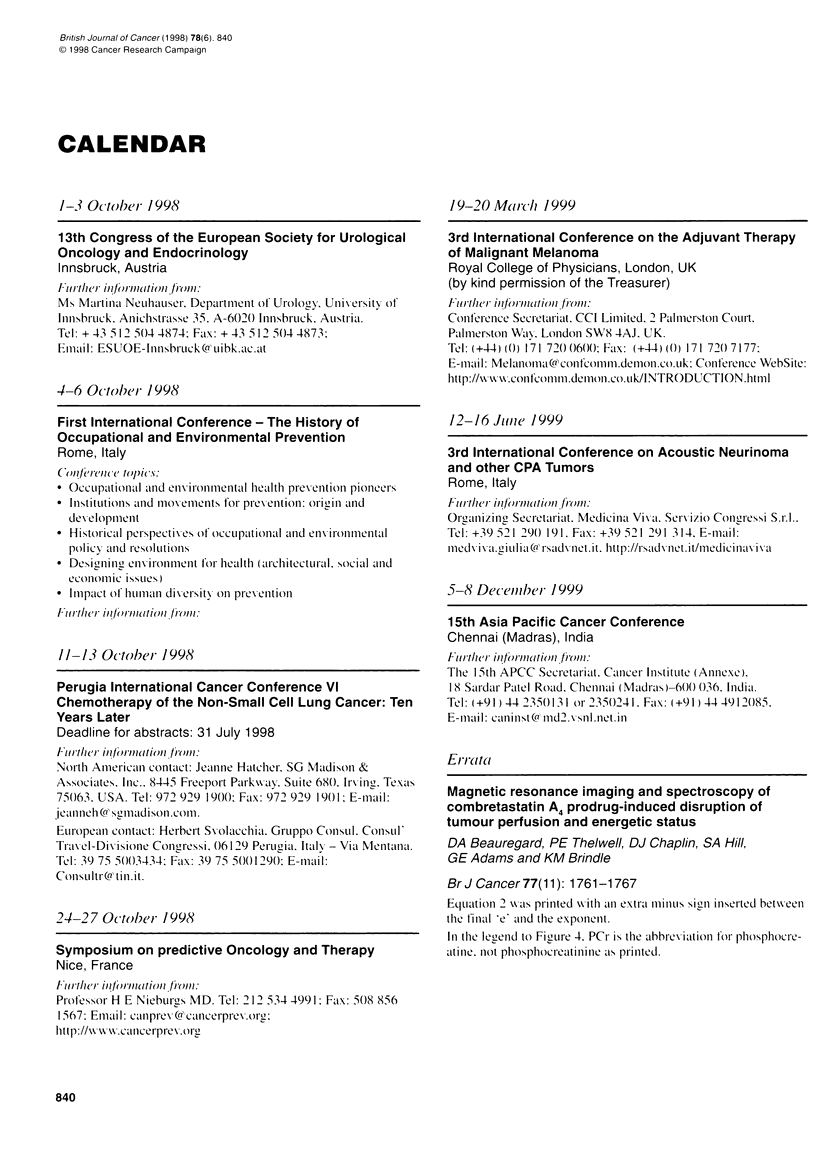# Calendar

**Published:** 1998-09

**Authors:** 


British Journal of Cancer (1998) 78(6), 840
© 1998 Cancer Research Campaign

### CALENDAR

### *1–3 October 1998*

**13th Congress of the European Society for Urological Oncology and Endocrinology**
Innsbruck, Austria

*Further information from:*

Ms Martina Neuhauser, Department of Urology, University of Innsbruck, Anichstrasse 35, A-6020 Innsbruck, Austria.

Tel: + 43 512 504 4874; Fax: + 43 512 504 4873;

Email: ESUOE-Innsbruck@uibk.ac.at

### *4–6 October 1998*

**First International Conference – The History of Occupational and Environmental Prevention**
Rome, Italy

*Conference topics:*

- Occupational and environmental health prevention pioneers

- Institutions and movements for prevention: origin and development

- Historical perspectives of occupational and environmental policy and resolutions

- Designing environment for health (architectural, social and economic issues)

- Impact of human diversity on prevention

*Further information from:*

### *11–13 October 1998*

**Perugia International Cancer Conference VI Chemotherapy of the Non-Small Cell Lung Cancer: Ten Years Later**
Deadline for abstracts: 31 July 1998

*Further information from:*

North American contact: Jeanne Hatcher, SG Madison & Associates, Inc., 8445 Freeport Parkway, Suite 680, Irving, Texas 75063, USA. Tel: 972 929 1900; Fax: 972 929 1901; E-mail: jeanneh@sgmadison.com.

European contact: Herbert Svolacchia, Gruppo Consul, Consul' Travel-Divisione Congressi, 06129 Perugia, Italy – Via Mentana. Tel: 39 75 5003434; Fax: 39 75 5001290; E-mail: Consultr@tin.it.

### *24–27 October 1998*

**Symposium on predictive Oncology and Therapy**
Nice, France

*Further information from:*

Professor H E Nieburgs MD. Tel: 212 534 4991; Fax: 508 856 1567; Email: canprev@cancerprev.org; http://www.cancerprev.org

### *19–20 March 1999*

**3rd International Conference on the Adjuvant Therapy of Malignant Melanoma**
Royal College of Physicians, London, UK
(by kind permission of the Treasurer)

*Further information from:*

Conference Secretariat, CCI Limited, 2 Palmerston Court, Palmerston Way, London SW8 4AJ, UK.

Tel: (+44) (0) 171 720 0600; Fax: (+44) (0) 171 720 7177;

E-mail: Melanoma@confcomm.demon.co.uk; Conference WebSite:

http://www.confcomm.demon.co.uk/INTRODUCTION.html

### *12–16 June 1999*

**3rd International Conference on Acoustic Neurinoma and other CPA Tumors**
Rome, Italy

*Further information from:*

Organizing Secretariat, Medicina Viva, Servizio Congressi S.r.l., Tel: +39 521 290 191, Fax: +39 521 291 314, E-mail: medviva.giulia@rsadvnet.it, http://rsadvnet.it/medicinaviva

### *5–8 December 1999*

**15th Asia Pacific Cancer Conference**
Chennai (Madras), India

*Further information from:*

The 15th APCC Secretariat, Cancer Institute (Annexe), 18 Sardar Patel Road, Chennai (Madras)–600 036, India.

Tel: (+91) 44 2350131 or 2350241, Fax: (+91) 44 4912085,

E-mail: caninst@md2.vsnl.net.in